# Beneficial effect of time-restricted eating on blood pressure: a systematic meta-analysis and meta-regression analysis

**DOI:** 10.1186/s12986-022-00711-2

**Published:** 2022-11-08

**Authors:** Weihao Wang, Ran Wei, Qi Pan, Lixin Guo

**Affiliations:** 1 Department of Endocrinology, Beijing Hospital, National Center of Gerontology, Institute of Geriatric Medicine, Chinese Academy of Medical Sciences, Beijing, People’s Republic of China; 2 Fifth School of Clinical Medicine, Peking University, Beijing, People’s Republic of China

**Keywords:** Time-restricted eating, Blood pressure, Meta-analysis

## Abstract

**Background:**

As a cardiometabolic disease, hypertension has shown an obvious upward trend, becoming a global epidemic chronic disease. Lifestyle intervention is a fundamental method for lowering blood pressure. This systematic review and meta-analysis aimed to evaluate the effects of time-restricted eating (TRE) on blood pressure.

**Methods:**

Studies were retrieved from the PubMed, Embase, Cochrane Library, and Web of Science databases to evaluate the effects of TRE on blood pressure. The time frame of search was from the start of database construction until July 14, 2022.There were no language restrictions. Meta-analysis and meta-regression were performed using Stata version 16. The weighted mean difference with 95% CI was used to assess the effect of TRE on blood pressure, heart rate, weight, blood glucose, total cholesterol, HDL-C, LDL-C, and triglycerides. The main ending of this article were blood pressure and heart rate, while the secondary ending were weight, blood glucose, total cholesterol, HDL-C, LDL-C, and triglycerides.

**Results:**

Ten randomized controlled trials involving 694 patients were identified. TRE significantly reduced systolic blood pressure (SBP) (mean difference = −4.15; 95% CI: −6.73, −2.30; *P* < 0.0001), but had no significant effect on diastolic blood pressure (DBP) (mean difference = −2.06; 95% CI: −4.16, 0.02; *P* = 0.053) and no beneficial effect on heart rate (mean difference = 0.36; 95% CI: −2.83, 3.54; *P* = 0.0825). TRE promoted weight loss (mean difference = −1.63; 95% CI: −2.61, −0.64; *P* = 0.001) and decreased blood glucose levels (mean difference = −2.80; 95% CI: −4.64, −0.96; *P* = 0.003), but had no significant effect on total cholesterol (mean difference = 0.03, 95% CI: −10.01, 10.08; *P* = 0.995), HDL-C (mean difference = 0.85, 95% CI: −1.80, 3.49; *P* = 0.531), LDL-C (mean difference = −0.86, 95% CI: −6.47, 4.76; *P* = 0.764), or triglycerides (mean difference = −3.524, 95% CI: −9.49, 2.45; *P* = 0.248). In a separate meta-regression analysis, the degree of SBP change was related to weight loss (*P* = 0.044) but not to glucose improvement (*P* = 0.867).

**Conclusions:**

The present meta-analysis suggests that TRE significantly reduced SBP, while no effect of reducing DBP was seen. The observed lower blood pressure may be attributed to significant weight loss. The effects of TRE on heart rate and blood lipid levels were not apparent.

## Background

On a global scale, modern humans face numerous complex chronic health challenges, such as obesity, diabetes, metabolic disease, and cardiometabolic disease. The presence of artificial light enables humans to remain active for 24 h a day. The chaotic activity-rest cycle indirectly disrupts the natural daily cycle of eating and fasting while unconsciously encouraging excess caloric intake. Chronically disrupted temporal regulation not only leads to metabolic disease, but also accelerates the aging process [1–3].

Recently, it has been suggested that intermittent and periodic fasting may be promising methods for optimizing longevity [4]. Fasting enables the body to enter alternative metabolic phases which are less dependent on glucose and more dependent on ketone body carbon sources. Nearly ten years ago, large-scale clinical trials [5–8] confirmed that intermittent fasting (IF) can reduce body weight and body fat, improve insulin sensitivity, reduce glucose and insulin levels, lower blood pressure (BP), improve lipid profiles, and reduce markers of inflammation and oxidative stress. The forms of IF investigated include alternate-day fasting, the 5:2 diet, and time-restricted eating (TRE). TRE, in which feeding times are restricted to specific times of the day, produced a similar effect to IF and conveyed benefits ranging from prevention to treatment of metabolic diseases. As a metabolic disease of the heart,, hypertension has shown an obvious upward trend, becoming a global epidemic chronic disease [9]. Lifestyle intervention is an essential method for lowering BP. However, thus far no systematic review or meta-analysis has directly linked TRE to BP . Therefore, the aim of this systematic review and meta-analysis was to evaluate the effects of TRE on BP.

## Methods

This meta-analysis was reported in accordance with the Preferred Reporting Items for Systematic Review and Meta-Analyses (PRISMA) guidelines.

### Search strategy

Based on the Population, Intervention, Comparator, Outcomes, and Study (PICOS) design framework, we searched the PubMed, Embase, Cochrane Library, and Web of Science databases to determine the effects of TRE on BP. Keywords, truncation symbols, medical subject heading (MeSH) terms, and Boolean operators (AND/OR) were used in the search strategy. The MeSH table retrieval formula was as follows: ‘time-restricted eating’ [MeSH] OR ‘blood pressure’ [MeSH]. The keyword search included the following terms: Time Restricted Feeding [Title/Abstract] OR Time Restricted Fasting [Title/Abstract] OR Intermittent Fasting [Title/Abstract] OR Fasting, Intermittent [Title/Abstract] OR Intermittent Fasting [Title/Abstract] OR Feeding, Time Restricted [Title/Abstract] OR Time Restricted Feedings [Title/Abstract] OR Ramadan [Title/Abstract] combined with Arterial Pressure [Title/Abstract] OR Hemodynamics [Title/Abstract] OR Systolic Blood Pressure (SBP) [Title/Abstract] OR Diastolic Blood Pressure (DBP) [Title/Abstract]. The most recent search was performed on July 14, 2022.

### Inclusion criteria and data extraction

Studies were included in our meta-analysis if (1) the participants included mostly adults with metabolic diseases such as obesity, (2) intervention involved limiting daily meal times to 4–12 hours, (3) A normal dietary eating strategy group was set as the control group, (4) the study outcomes/metrics included BP, including baseline and post-intervention values, expressed as the mean and standard deviation (SD), (5) Priority was given to randomized control trials (RCT), but observational studies were also accepted, provided they included a control group without TRE. Articles were excluded based on the following criteria: (1) animal experiments rather than human adult studies; (2) editorials, letters, reviews, commentaries, or interviews; (3) lack of control group, such as only one experimental group; (4) the intervention method was not TRE; (5) BP values were incomplete.

Using the aforementioned inclusion/exclusion criteria, two independent researchers (Weihao Wang and Ran Wei) reviewed the titles and abstracts of each retrieved paper. If there were any uncertainties regarding qualifications, a third researcher studied the full text. A consensus was reached for all studies.

### Data collection and registered protocols

Potential eligible articles were collected based on the inclusion and exclusion criteria. The following data were acquired from the selected articles: data source and setting, study design, participants, study duration, TRE regimen (Fasting: Feeding), total number, age, sex, body mass index (BMI), and indicators, including SBP, DBP, weight, glucose, total cholesterol, triglycerides, low-density lipoprotein cholesterol (LDL-C), and high-density lipoprotein cholesterol (HDL-C).

Because this study collected data from articles published by others for reporting, no ethics approval was required. We registered the systematic review and meta-analysis protocols at inplasy.com under the registration number Inplasy Protocol 202280057 (https://doi.org/10.37766/inplasy2022.8.0057).

### Statistical analysis

Meta-analysis and meta-regression were performed using Stata version 16 (StataCorp, College Station, TX, USA). The weighted mean difference with 95% CI was used to assess the effect of TRE on BP, heart rate, weight, blood glucose, cholesterol, HDL-C, LDL-C, and triglycerides. We used the I^2^ index to quantify statistical heterogeneity. I^2^>50% indicated apparent heterogeneity, and <50% indicated no apparent heterogeneity. A random-effects model was used if apparent heterogeneity was observed. Otherwise, a fixed effects model was used. Subgroup analyses were performed to identify sources of heterogeneity. A sensitivity analysis was performed by individually excluding documents to assess the stability of the results. Publication bias was evaluated using a filled funnel plot. Egger’s test was employed to assess the probability of publication bias at a significance level of 10%. We used the GRADE software to evaluate the quality of evidence and evaluated the quality of the included studies using the risk of bias tool in Revman 5.4.

## Results

### Literature search

**Fig. 1:**
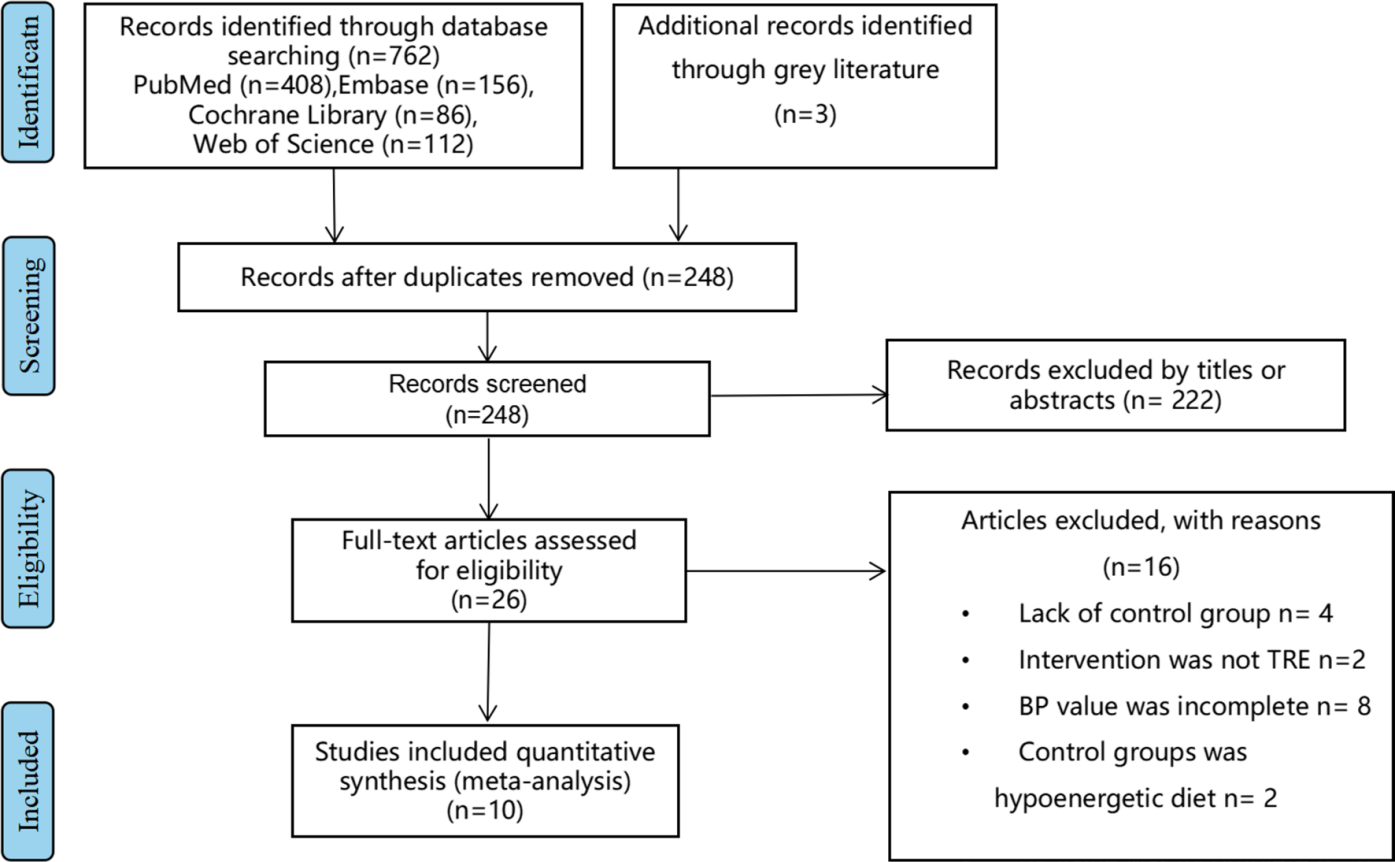
Flowchart of the study selection

A total of 762 articles were retrieved (408 from PubMed, 86 from the Cochrane Library, 112 from the Web of Science, and 156 from Embase), of which 26 articles met the inclusion criteria. Sixteen articles were further excluded: 4 lacked a normal eating dietary strategy group [10–13]; in 2 articles, the intervention method was not TRE [14, 15]; in 8 articles, the BP value was incomplete [16–23]; and in 2 articles, the control group was a hypoenergetic diet [24, 25]. Therefore, 10 eligible studies [26–35] were included in the final meta-analysis. Of these, reference 24 included three groups, with two different TRE intervention groups. A flowchart of the study selection process is shown in Fig. 1.

### Study characteristics

The study characteristics are listed in Table 1, which includes data source and setting, study design, participants, study duration, TRE regimen (Fasting: Feeding), total number, age, sex, and BMI.

**Table 1:** Characteristics of the studies investigating the effects of Time-Restricted Eating on blood pressure

Reference	Study design	Participants	Study duration	TRE regimen (fasting: feeding)	Total number	Age (year)	Sex: Male (%)	BMI (kg/m ^2^ )
Cienfuegos [26]	RCT	Obesity	8 weeks	4-h TRF (20:4), 6-h TRF (18:6), or a control group	58	47 [3]	8.6	37 [1]
Phillips [27]	RCT	Metabolic syndrome	6 months	12 h time-restricted eating (12:12), standard dietary advice	213	40.1 (13.3)	28.6	24.9 (22.6-29.1)
Gabel [28]	–	Obese adults	12 weeks	8-h time restricted feeding(16:8) , matched historical control group	46	50 [2]	89.1	35 (1)
Aliasghari [29]	Observational trial	Nafld patients	–	fast for Ramadan(16:8) ,not to fast for Ramadan	83	37.59 (7.06)	68.7	30.09 (4.49)
Dewanti [30]	–	Male outdoor workers	1 month	fast for Ramadan(16:8) ,not to fast for Ramadan	100	–	100	24.2 (3.2)
Lowe [31]	RCT	Overweight and obesity	12 weeks	8-h time restricted feeding(16:8),consistent meal timing group	50	43.8 (11.2)	56.0	31.4 (4.0)
Chow [32]	–	Overweight	12 weeks	TRE (8-hour window), non-TRE(unrestricted eating)	20	46.5 (12.4)	15	33.8 (7.6)
Kotarsky [33]	RCT	Overweight and obese adults	8 weeks	TRE consumed all calories between 12:00 p.m. and 8:00 p.m(16:8) ,normal eating (NE) dietary strategy group	21	45 (3)	14.3	29.8 (0.8)
Tinsley [34]	RCT	Active females	8 weeks	TRF(~7.5 h/d),control diet	40	22.0 (2.4)	0	–
Lin [35]	Randomized,open-label,parallel-group design,	Middle-aged women	8 weeks	TRF group (limit 8 h of eating time and fasting for 16 h) , a non-TRF group	63	50.1 (7.5)	0	25.9 (3.7)

Age and BMI values are expressed as mean ± SD.BMI, body mass index; RCT,randomized controlled trial;NAFLD, nonalcoholic fatty liver disease

### Main outcomes

**Fig. 2:**
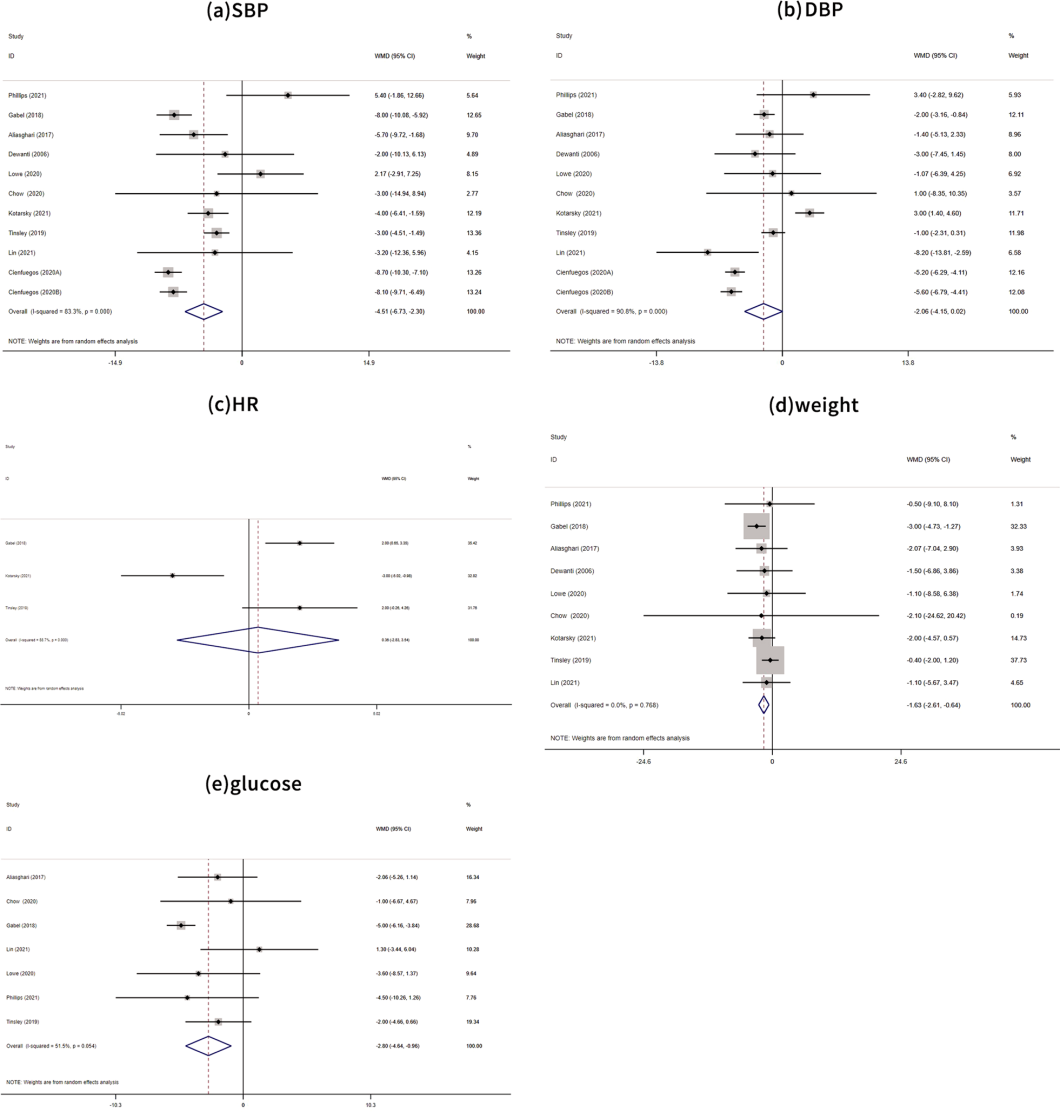
Forest plots of TRE vs. normal dietary eating in overall analyses, and **a** based on SBP changes **b** based on SBP changes **c** based on heart rate changes; **d** based on weight changes; and **e** based on blood glucose. TRE, time-restricted eating; SBP, systolic blood pressure

A meta-analysis of ten studies [26–35] showed that TRE significantly reduced SBP (mean difference = −4.15, 95% CI: −6.73, −2.30; *P* < 0.0001), as shown in Fig. 2a. Meanwhile, TRE had no significant effect on DBP (mean difference = −2.06, 95% CI: −4.16, 0.02; *P* = 0.053), as shown in Fig. 2b. Although 10 studies were included, the graphs have 11 rows because reference 24 includes three groups, including two different TRE intervention groups.

### Secondary outcomes

A meta-analysis of three studies [28, 33, 34] showed that TRE was unable to lower the heart rate (mean difference =0.36, 95% CI: −2.83, 3.54; *P* = 0.825), as shown in Fig. 2c.

The results of a meta-analysis of nine [27–35] studies showed that TRE significantly reduced weight (mean difference = −1. 63, 95% CI: −2.61, −0.64; *P* = 0.001), as shown in Fig. 2d.

A meta-analysis of seven studies [27–29, 31, 32, 34, 35] showed that TRE significantly reduced blood glucose levels (mean difference = −2.80, 95% CI: −4.64, −0.96; *P* = 0.003), as shown in Fig. 2e.

**Fig. 3:**
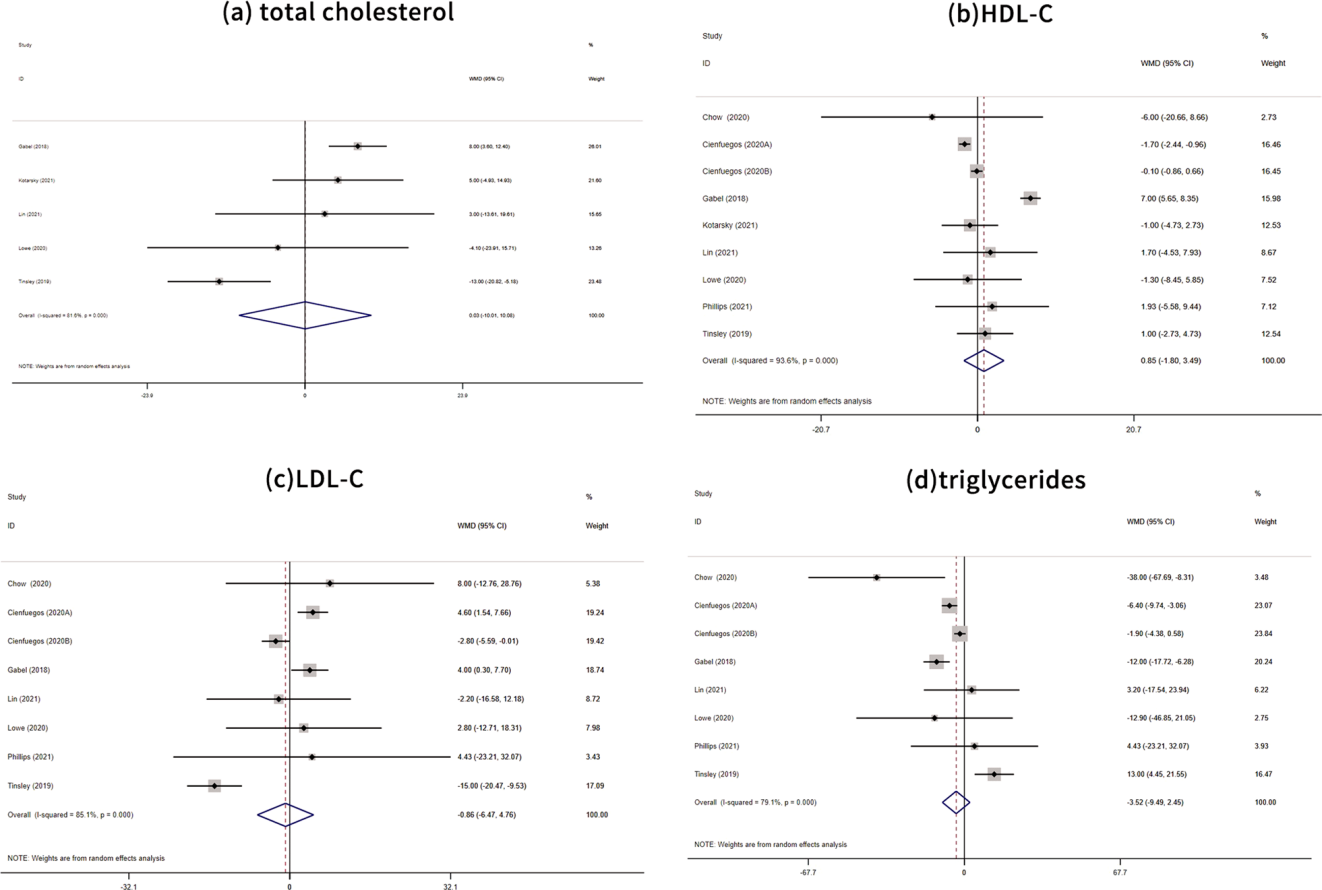
Forest plots of TRE vs. normal eating dietary on blood lipids **a** based on cholesterol levels; **b** based on HDL-C; **c** based on LDL-C; and **d** based on triglycerides. *TRE* time-restricted eating; *HDL-C* high-density lipoprotein cholesterol; *LDL-C* low-density lipoprotein cholesterol

A meta-analysis of five studies [28, 31, 33–35] showed that TRE had no significant effect on total cholesterol (mean difference = 0.03, 95% CI: −10.01, 10.08; *P* = 0.995), as shown in Fig. 3a. A meta-analysis of eight studies [26–28, 31–35] showed that TRE insignificantly increased HDL-C (mean difference = 0.85, 95% CI: −1.80, 3.49; P = 0.531), as shown in Fig. 3b. A meta-analysis of seven studies [26–28, 31, 32, 34, 35] showed that TRE decreased LDL-C (mean difference = −0.86, 95% CI: −6.47, 4.76; *P* = 0.764), as shown in Fig. 3c. A meta-analysis of seven studies [26–28, 31, 32, 34, 35] showed that TRE decreased triglyceride levels (mean difference = −3.52, 95% CI: −9.49, 2.45; *P* = 0.248), as shown in Fig. 3d.

### Subgroup analysis

As shown in Table 2, upon stratification by the duration of TRE intervention, we divided the studies into two groups 8 weeks (n = 3) and 12 weeks (n = 4). DBP was significantly reduced in patients with an intervention time of 12 weeks (WMD = −1.916 mmHg, 95% CI: −3.037, −0.794, *P* = 0.001), with low heterogeneity (I^2^= 0.0%).

**Table 2:** Subgroup Analysis. SBP, systolic blood pressure; DBP, diastolic blood pressure; WMD, weighted mean difference; 95% CI, 95% confidence interval

Index	Subgroup (weeks)	Num. of trials	WMD	95% CI	*P*	I^2^ (P)
SBP	12	4	−3.197	−11.140 to 4.745	0.430	85.2% (0.001)
	8	3	−5.794	−8.610 to −2.979	0.000	88.7% (0.000)
DBP	12	4	−1.916	−3.037 to−0.794	0.001	0.0% (0.782)
	8	3	−3.054	−6.422 to 0.314	0.076	96.0% (0.000)

### Sensitivity analysis

As shown in Table 3, sensitivity analysis was performed on nine indicators, including SBP, DBP, heart rate, weight, glucose, total cholesterol, HDL-C, LDL-C, and triglycerides. After excluding studies individually, the combined effect size before and after did not change significantly, indicating that the results of the meta-analysis were relatively stable.

**Table 3:** Sensitivity Analysis. SBP, systolic blood pressure; DBP, diastolic blood pressure; HR,heart rate;95% CI, 95% confidence interval

Index	Effect	95%CI lower	95% CI upper	Change 95% CI lower	Change 95% CI upper
SBP	−4.51	−6.73	−2.30	−7.36	−1.39
DBP	−2.06	−4.15	0.02	−4.6	0.63
HR	0.36	−2.8	3.54	−5.43	4.46
Weight	−1.63	−2.61	−0.64	−3.62	0.23
Glucose	−2.80	−4.64	−0.96	−5.07	−0.31
Total cholesterol	0.03	−10.01	10.08	14.58	11.92
HDL-C	0.85	−1.80	3.49	−3.25	4.86
LDL-C	−0.86	−6.47	4.76	−8.64	7.15
Triglycerides	−3.52	−9.49	2.45	−13.29	5.63

### Meta-regression

We included nine studies that included changes in body weight and seven studies that included changes in blood glucose levels using meta-regression. The random-effect meta-regression of the primary meta-analysis on SBP revealed that body weight change (*P* = 0.044) predicted the size of the estimated treatment effect or explained heterogeneity between studies, while glucose (*P* = 0.867) did not. We further found that weight loss can predict TRE-induced SBP reduction.

### Funnel plots and egger tests

**Fig. 4:**
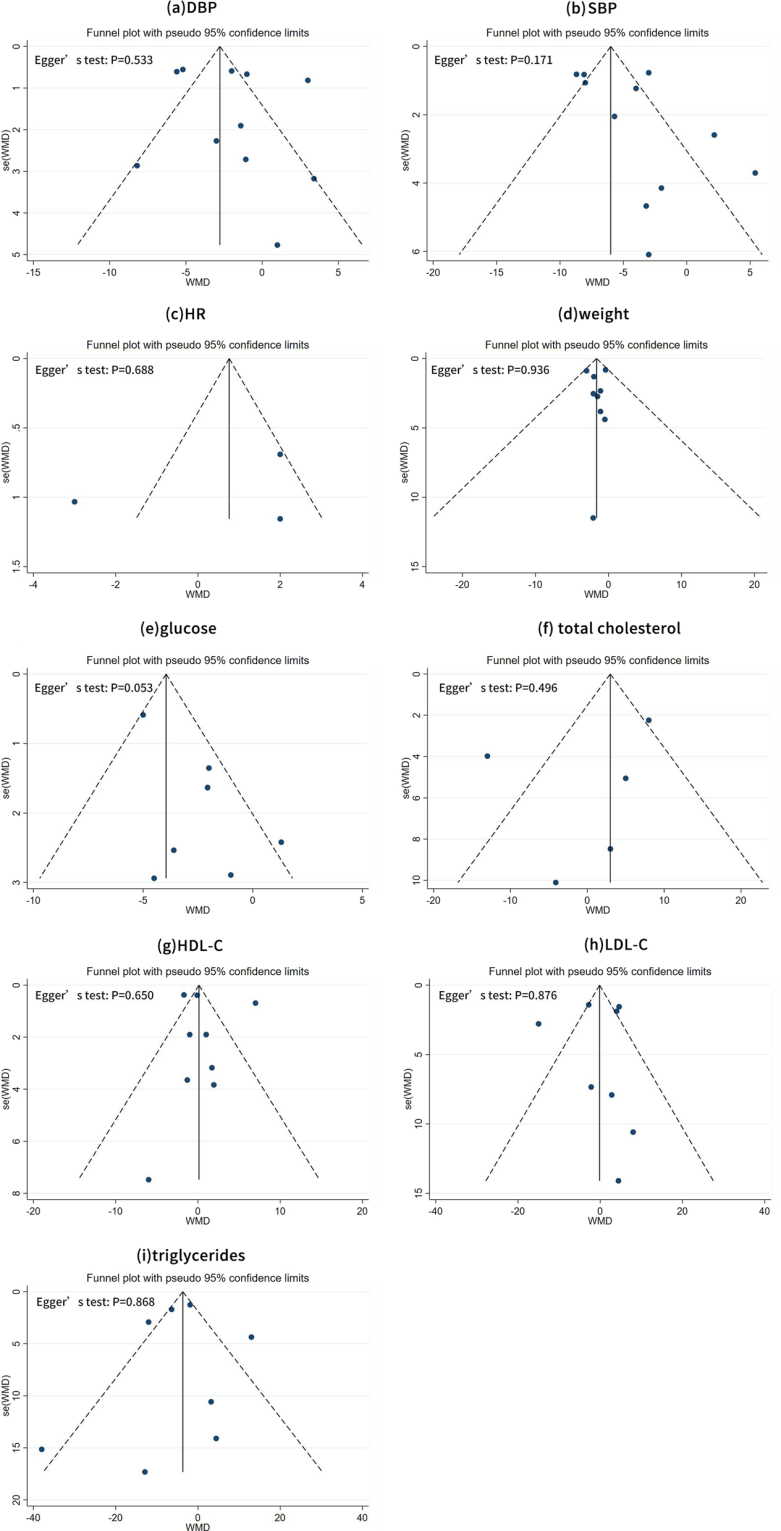
Filled funnel plots of TRE vs. normal dietary eating **a** based on SBP changes; **b** based on SBP changes; **c** based on heart rate changes; **d** based on weight changes; **e** based on blood glucose levels; **f** based on cholesterol; **g** based on HDL-C; **h** based on the LDL-C; **i** based on triglycerides. *TRE* time-restricted eating; *SBP* systolic blood pressure; *HDL-C* high-density lipoprotein cholesterol; *LDL-C* low-density lipoprotein cholesterol

As reflected by the filled funnel plots (Fig. 4), there was obvious heterogeneity in the SBP (Fig. 4b). In addition, as indicated by Egger’s tests, there was a low probability of publication bias for all indexes under study (all *P* > 0.05).

### The quality of evidence assessment

**Fig. 5:**
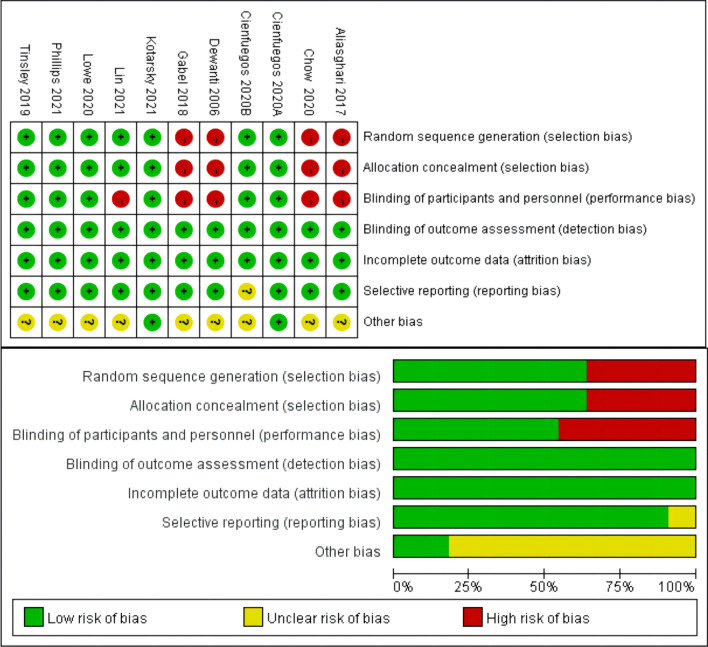
The quality of evidence assessment

Ten studies included in our analysis were assessed for their quality. As shown in Fig. 5, we evaluated the quality assessment of the included studies using the risk of bias tool in Revman 5.4.

## Discussion

Unlike previous studies, which focused on body weight and blood glucose levels, this meta-analysis primarily focused on the effects of TRE on BP. To the best of our knowledge, this is the first meta-analysis to investigate this relationship. The results of a meta-analysis of ten studies showed that TRE significantly reduced SBP. Furthermore, this relationship remained consistent across the subgroup analyses. Meanwhile, TRE significantly reduced DBP in patients with an intervention duration of 12 weeks. These findings are critical because they provide a new perspective on decreasing BP.

In recent years, TRE, a weight loss method, has shown enormous promise in fighting obesity and metabolic diseases. The concept of TRE stems from studies of the effects of food timing on the circadian system. One of the major adverse consequences of circadian rhythm disturbances is an increased risk of cardiovascular diseases [36]. Cardiovascular diseases remain a leading cause of death globally in the general population; consequently, there is growing interest in the circadian regulation of cardiovascular health.

However, TRE was shown to have little beneficial effect on heart rate. Therefore, lower BP may be attributed to distinct weight loss. As is well known, there is a strong link between body weight and BP in obese patients [37]. Several mechanisms may lead to hypertension, such as insulin and leptin resistance, perivascular adipose tissue dysfunction, renal impairment, renin-angiotensin-aldosterone activation, and sympathetic nervous system activity [37]. Weight loss has beneficial effects on BP. In this study, we further analyzed the effect of TRE on blood lipid levels (total cholesterol, triglycerides, LDL-C, and HDL-C). Unfortunately, we found no obvious effects in our study. Recent studies [38] using metabolomics and lipidomics platforms have shown that the levels of hundreds of lipid species in the plasma are regulated by circadian rhythms, although the timing and magnitude of the lipid rhythms vary widely among individuals. A prior meta-analysis by Chen et al. [39] demonstrated that LDL-C levels were increased in the TRE group, which may partially explain our poor results.

Broadly speaking, the results of previous animal experimentation were consistent with the conclusions of the present study. An animal model conducted by Cote et al. [40] illustrated that limiting feeding in the active phase reduces BP. Hou et al. demonstrated [41] that TRE protects the circadian rhythm of BP in db/db mice by suppressing sympathetic activity during the light phase. Furthermore, Mager et al. [42] demonstrated that intermittent fasting reduces heart rate in rats. The increased variability in heart rate in rats caused by TRE may result from the enhanced activity of brainstem cholinergic cardiovagal neurons [43]. Godar et al. [44, 45] showed that intermittent fasting improved myocardial ischemia-reperfusion injury and reduced circulating cholesterol and triglyceride levels. In general, animal studies have demonstrated significant cardioprotective effects of TRE.

Our study has several limitations which should be mentioned. Firstly, as shown in Table 1, the vast majority of our subjects were overweight or obese, while patients with hypertension were excluded from the present study. Therefore, it is prudent to conclude that TRE improves BP in obese individuals. Whether this conclusion can be extended to hypertensive populations requires further investigation. Secondly, the heterogeneity of the studies was significant; this was attributed to the different time ranges and durations of the TRE interventions. The longest intervention period in the included studies lasted 12 months, whereas the shortest was 1 month. These factors may have had different degrees of influence on the results. Despite this, our results remained statistically significant. Furthermore, compared with DBP, TRE significantly reduced the SBP. Heart rate and peripheral resistance had a significant effect on DBP. This appears to explain the DBP changes with inconspicuous improvement in heart rate. At the same time, the included participants were generally younger with higher peripheral resistance; therefore, the changes in DBP were not distinct. In addition, intervention components such as calorie restriction or exercise are thought to influence BP, weight, lipids, and glucose. Kotarsky et al. [33] reported that participants in both the TRE and normal eating groups completed eight weeks of aerobic exercise and supervised resistance training. As both the experimental and control groups exercised, we excluded the liability of exercise on BP, weight, lipids, and glucose. Finally, the effect of different TRE restriction times (e.g. 4 h, 6 h, 8 h, 12 h) would have had an effect on the outcome. However, owing to the number of original studies, the TRE restriction time could not be refined further. Our results need to be validated by RCT studies with large samples and long-term follow-up.

There is already a wealth of evidence, both in the basic and clinical fields, to suggest the effects of TRE. However, there is still room for improvement in the following aspects. First, the effects of TRE on the heart rate and blood lipid levels were not apparent in our study, which was not consistent with the results of basic research. More convincing data on the effects of TRE on heart rate and blood lipids are still required. Simultaneously, we generalized our conclusions to patients with prehypertension and hypertension. As a simple and accessible means, TRE promises to improve the lifestyle of prehypertensive and hypertensive patients.

## Conclusions

The present meta-analysis suggests that TRE significantly reduced SBP while the effect of reducing DBP was not obvious. The lower BP may be attributed to the significant weight loss. The effects of TRE on heart rate and blood lipid levels were not apparent. There is an urgent need for higher quality RCTs with a longer follow-up time to prove our results.

## Abbreviations

IF: Intermittent fasting
TRE: Time-restricted eating
BP: Blood pressure
PRISMA: Preferred Reporting Items for Systematic Review and Meta-Analyses
PICOS: Population, Intervention, Comparator, Outcomes, and Study
SBP: Systolic Blood Pressure
DBP: Diastolic Blood Pressure
SD: Standard deviation
LDL-C: Low-density lipoprotein cholesterol
HDL-C: High-density lipoprotein cholesterol
BMI: Body mass index
MeSH: Medical subject heading
RESET: REStricted Eating Time
